# Adipose tissue area is a predictive biomarker for the efficacy of pegylated liposomal doxorubicin in platinum‐refractory/resistant ovarian cancer

**DOI:** 10.1002/cam4.6086

**Published:** 2023-05-15

**Authors:** Hiroyuki Yoshida, Keiichi Fujiwara

**Affiliations:** ^1^ Department of Gynecologic Oncology Saitama Medical University International Medical Center Hidaka Saitama Japan

**Keywords:** adipose tissue, ovarian cancer, pegylated liposomal doxorubicin, predictive biomarker

## Abstract

**Background:**

Pegylated liposomal doxorubicin (PLD), an anthracycline agent, is widely used as a treatment option for platinum‐refractory/resistant epithelial ovarian cancer (EOC). Although only a subset of patients with platinum‐refractory/resistant EOC derive benefit from PLD, predictive biomarkers for patients who will respond to the drug have not yet been established. Here, we evaluated the relationship between adipose tissue status and PLD efficacy in patients with platinum‐refractory/resistant EOC.

**Methods:**

Patients with platinum‐refractory/resistant EOC who were treated with single‐agent PLD were included in this retrospective cohort study. Adipose tissue areas including visceral adipose tissue area (VATA), subcutaneous adipose tissue area (SATA), and visceral to subcutaneous adipose tissue area ratio (VSR) were calculated prior to the initiation of PLD using computed tomography images. The associations of adipose tissue areas with objective response rate (ORR) and patient survival were evaluated.

**Results:**

Forty‐four patients with platinum‐refractory/resistant EOC who received single‐agent PLD were included. Subjects were categorized into high and low groups according to the median VATA, SATA, and VSR values, and body mass index (BMI). The ORR of PLD was significantly lower in the VSR‐high group than in the VSR‐low group (*p =* 0.0089). Patients in the high VSR group showed significantly shorter progression‐free survival (PFS) compared with patients in the low VSR group (median, 4.0 vs. 8.5 months; *p* = 0.020). In the multivariable analysis, high VSR was a significant prognostic factor for shorter PFS (hazard ratio, 2.07; 95% confidence interval, 1.05–4.19; *p* = 0.035). VATA, SATA, and BMI showed no significant association with ORR and survival of patients who received PLD.

**Conclusions:**

High VSR is associated with lower ORR and shorter PFS in patients with platinum‐refractory/resistant EOC who received single‐agent PLD. VSR is a robust predictive biomarker for the efficacy of PLD.

## 1 INTRODUCTION

Epithelial ovarian cancer (EOC) has the poorest prognosis of all the gynecologic malignancies.^1^ Because there is no effective screening method for EOC, many cases are classified as advanced cancer.^2^ Initial therapy for advanced EOC includes surgery followed by taxane and platinum‐based chemotherapy.^3^, ^4^ Approximately 70% of EOC cases respond to initial treatment, but most advanced EOC cases recur.^5^, ^6^, ^7^ EOC that relapses within 4 weeks of treatment with platinum‐based therapy is defined as platinum‐refractory, and those that relapse within 1 to 6 months of treatment are classed as platinum‐resistant. Platinum‐refractory/resistant EOC responds poorly to most chemotherapies and generally has a poor prognosis.^8^ Establishment of effective treatment for platinum‐refractory/resistant EOC is an urgent issue in gynecologic cancer treatment.

Pegylated liposomal doxorubicin (PLD) is widely administered to platinum‐refractory/resistant EOC patients.^9^ However, the response rate of platinum‐refractory/resistant EOC to PLD is not high (approximately 10%–20%), while PLD causes a variety of adverse events including cardiotoxicity and hand‐foot syndrome.^9^, ^10^, ^11^, ^12^ Because only a subset of patients with platinum‐refractory/resistant EOC respond to PLD, predicting which patients will benefit from PLD is critical to avoid ineffective chemotherapy and unnecessary adverse events. Therefore, a biomarker that predicts the efficacy of PLD is strongly needed in the clinical practice of gynecologic oncology. However, biomarkers of PLD efficacy in platinum‐refractory/resistant EOC patients have not yet been established.

Adipose tissue contributes to the resistance of cancers to various anticancer agents. It has been reported that visceral adipose tissue attenuates tumor response to chemotherapy in breast cancer,^13^ and furthermore, visceral adipose tissue has been shown to be a poor prognostic factor in breast cancer patients treated with neoadjuvant chemotherapy.^14^ On the contrary, in liver cancer, visceral adipose tissue has been reported to be involved in chemotherapy resistance.^15^ One possible mechanism by which adipose tissue is associated with chemotherapy resistance is that adipose tissue secretes various growth factors, including angiogenic factors. It has been reported that angiogenic factors from adipose tissue bind and neutralize the antiangiogenic agents and attenuate their effects.^16^, ^17^ In ovarian cancer patients, adipose tissue has also been reported to be associated with the efficacy of bevacizumab, an antiangiogenic agent.^18^ However, there are few reports of adipose tissue being associated with the efficacy of anticancer agents in ovarian cancer.

Furthermore, adipose tissue is involved in resistance to anthracyclines. It has been demonstrated that adipose tissue attenuates the effect of anthracyclines (daunorubicin and doxorubicin) by absorbing and inactivating them.^19^, ^20^ In addition, adipose tissue has been shown to increase the expression of major vault protein (MVP) in cancer cells, causing resistance to doxorubicin, an anthracycline.^21^ Because PLD is an anthracycline, adipose tissue may also be involved in the resistance to PLD. However, to date, no report has examined whether adipose tissue can be a predictive biomarker of PLD efficacy. Thus, we present the first analysis investigating whether adipose tissue could be a predictive biomarker of PLD efficacy in platinum‐refractory/resistant EOC patients.

## 2 MATERIALS AND METHODS

### 2.1 Study design and patient selection

This single institution‐based retrospective cohort study was approved by the Institutional Review Board of Saitama Medical University International Medical Center (reference number: 20‐096). Because this was a retrospective study, informed consent was waived. This study was carried out in accordance with the Declaration of Helsinki.

Patients with histologically confirmed EOC, fallopian tube cancer, or peritoneal cancer treated with single‐agent PLD between January 2013 and December 2019 at our institution were included. The follow‐up period ended on December 31, 2021. The inclusion criteria were as follows: (1) patients >18 years of age at diagnosis; (2) patients diagnosed with platinum‐resistant or platinum‐refractory epithelial ovarian, fallopian tube, or peritoneal cancer; and (3) patients with available abdominal computed tomography (CT) scans undertaken no more than 1 month before PLD treatment began. The exclusion criteria were as follows: (1) patients with malignancies other than EOC; (2) patients who received PLD together with other anticancer agents; (3) patients treated with PLD for two or fewer cycles; and (4) patients with insufficient clinical data for analysis.

### 2.2 PLD treatment

PLD was administered by 1‐h intravenous infusion every 4 weeks at varying doses of 40 or 50 mg/m^2^. The recommended dose of PLD when given as monotherapy is 50 mg/m^2^, although reducing the dose to 40 mg/m^2^ does not change the efficacy.^22^, ^23^ Therefore, choice of PLD dose was left to the attending physician, taking into consideration each patient's age, general condition, and comorbidities. Treatment was given until disease progression was observed or until adverse events made continuation of treatment difficult.

### 2.3 Adipose tissue analyses

CT scans at the level of the umbilicus that were taken a month prior to treatment initiation of PLD were used to calculate visceral adipose tissue area (VATA) and subcutaneous adipose tissue area (SATA) by the Volume Analyzer SYNAPSE VINCENT 3D image analysis system (FUJIFILM Medical). Tissue Hounsfield unit thresholds were set as follows: −190 to −30 for subcutaneous adipose tissue and −150 to −50 for visceral adipose tissue.^24^ We estimated the visceral to subcutaneous adipose tissue area ratio (VSR), an indicator of intra‐abdominal visceral adipose tissue accumulation.^25^, ^26^ Body mass index (BMI) was also measured by dividing weight (in kilograms) by the square of height (in meters) immediately prior to the initiation of PLD treatment.

### 2.4 Evaluation of tumor response and survival

Investigators evaluated tumor response using a CT scan every 3 cycles of PLD treatment and the best overall response was assessed using the Response Evaluation Criteria for Solid Tumors (RECIST) version 1.1,^27^ which includes complete response, partial response, stable disease, and progressive disease. We measured the objective response rate (ORR) as the percentage of patients who achieved a complete response or partial response. Progression‐free survival (PFS) was calculated from the date of the initial PLD treatment until the first recorded evidence of progression or death from any cause. Patients with no disease progression were censored at the last follow‐up date. Overall survival (OS) was defined as the time from the start date of PLD treatment until death from any cause. Survivors were censored at the last follow‐up date. The follow‐up period ended on December 31, 2021.

### 2.5 Statistical analyses

Continuous variables are expressed as the means ± standard deviation or median (interquartile range [IQR]). Patients were categorized into high and low groups according to the median values of VATA, SATA, VSR, and BMI. We compared patients' characteristics, ORR, and survival between the high and low groups. We used chi‐square test or Fisher's exact test for comparisons of categorical variables. PFS and OS curves were constructed using the Kaplan–Meier method. Comparisons of PFS and OS were examined using the log‐rank test. Multivariable analysis of PFS and OS was conducted with Cox proportional hazard model and the results are expressed as hazard ratios (HRs) with 95% confidence intervals (CIs). Models were planned to adjust for age (< 65 or ≥65), performance status (0 or ≥1), stage (I/II or III/IV), histological type (serous/endometrioid or others), previous chemotherapy regimens (≤2 or >2), and platinum status (refractory or resistant). Statistical significance was defined as *p* < 0.05. Statistical analysis was performed using JMP Pro 13 software (JMP Pro 13, SAS Institute).

## 3 RESULTS

### 3.1 Patient inclusion and characteristics

**FIGURE 1:**
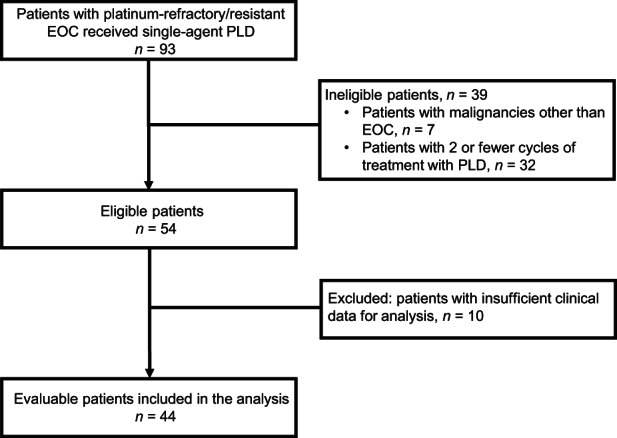
Patient inclusion flowchart. Ninety‐three patients with platinum‐refractory/resistant EOC received single‐agent PLD. Of these, 39 patients were excluded because they were not eligible for this study, and 10 patients were excluded because of insufficient clinical data for the analysis. In total, 44 patients were included in this study. EOC, epithelial ovarian cancer; PLD, pegylated liposomal doxorubicin.

Between January 2013 and December 2019, 93 patients with platinum‐refractory/resistant EOC received single‐agent PLD treatment. Of these, 39 patients were excluded due to ineligibility for this study, and 10 patients were excluded because of insufficient clinical data for analysis. As a result, 44 patients were included in this study (Figure 1). At the data cutoff (December 31, 2021), the median follow‐up was 11.5 months (IQR 6.0–26.0 months). Table 1 shows patient characteristics. The mean patient age was 60.7 ± 8.4 years. Fifty percent of the patients had stage III disease, and two‐thirds of the histological types were high‐grade serous carcinoma (65.9%). There were equal numbers (*n* = 22) of platinum‐refractory and platinum‐resistant patients. The median number of PLD administered cycles was 5.0 (IQR 3.0–9.7 cycles). The median BMI, VATA, SATA, and VSR values were 21.6 kg/m^2^ (IQR 19.3–23.6 kg/m^2^), 53.6 cm^2^ (IQR 34.8–82.0 cm^2^), 134.6 cm^2^ (IQR 78.6–191.1 cm^2^), and 0.44 (IQR 0.31–0.57), respectively.

**TABLE 1:** Patients' characteristics.

Characteristic	*n* = 44
Age (years)	60.7 (±8.4)
ECOG PS	
0	43 (97.7%)
≥1	1 (2.3%)
FIGO stage	
I	4 (9.1%)
II	4 (9.1%)
III	22 (50%)
IV	14 (31.8%)
Histological type	
High‐grade serous	29 (65.9%)
Endometrioid	4 (9.1%)
Clear cell	6 (13.6%)
Mixed	2 (4.6%)
Undifferentiated carcinoma	3 (6.8%)
Tumor origin	
Ovary	38 (86.4%)
Fallopian tube	3 (6.8%)
Peritoneum	3 (6.8%)
Platinum status	
Refractory	22 (50%)
Resistant	22 (50%)
Previous chemotherapy regimens	
≤2	25 (56.8%)
>2	19 (43.2%)
Primary cytoreductive surgery	25 (56.8%)
Interval cytoreductive surgery	19 (43.2%)
Residual disease	
<1 cm	34 (77.3%)
≥1 cm	10 (22.7%)
PLD dose	
40 mg/m^2^	24 (54.5%)
50 mg/m^2^	20 (45.5%)
Administered cycles of PLD	5.0 (3.0–9.7)
BMI (kg/m^2^)	21.6 (19.3–23.6)
VATA (cm^2^)	53.6 (34.8–82.0)
SATA (cm^2^)	134.6 (78.6–191.1)
VSR	0.44 (0.31–0.57)

*Note*: Data are shown as *n* (%) or mean (±standard deviation) or median (interquartile range).

Abbreviations: BMI, body mass index; ECOG PS, Eastern Cooperative Oncology Group performance status; FIGO, International Federation of Gynecology and Obstetrics; PLD, pegylated liposomal doxorubicin; SATA, subcutaneous adipose tissue area; VATA, visceral adipose tissue area; VSR, visceral to subcutaneous adipose tissue area ratio.

### 3.2 Tumor response analyses

The best overall response of the patients with platinum‐refractory/resistant EOC received single‐agent PLD was partial response in 7 (15.9%), stable disease in 21 (47.7%), and progressive disease in 16 (36.4%), while no patients with complete response were observed. Thus, the ORR of PLD in all patients was 15.9%. We examined whether clinical parameters such as age and histology, in addition to adipose tissue areas, were associated with the ORR of PLD. The ORR of PLD was significantly lower in the VSR‐high patient group than in the VSR‐low patient group (*p =* 0.0089; Table 2). Other clinical parameters did not significantly correlate with the ORR of PLD.

**TABLE 2:** Overall response rate of PLD and patients' characteristics.

Characteristic	No. of patient	ORR (95% CI)	*p* value
Age			0.68
<65	25	20.0% (8.4%–39.5%)	
≥65	19	10.5% (1.7%–32.6%)	
FIGO stage			0.59
I/II	8	25.0% (6.3%–59.9%)	
III/IV	36	13.8% (5.6%–29.1%)	
Histological type			0.34
Serous/endometrioid	33	12.1% (4.2%–27.9%)	
Others^a^	11	27.2% (9.2%–57.1%)	
Previous chemotherapy regimens			0.12
≤2	25	24.0% (11.1%–43.7%)	
>2	19	5.2% (−0.9%–26.4%)	
Platinum status			0.41
Refractory	22	22.7% (9.7%–43.8%)	
Resistant	22	9.1% (1.3–29.0%)	
BMI			1.0
Low	22	13.6% (3.9%–34.1%)	
High	22	18.1% (6.75–39.1%)	
VATA			1.0
Low	22	13.6% (3.9%–34.1%)	
High	22	18.1% (6.7%–39.1%)	
SATA			0.41
Low	22	9.0% (1.3%–29.0%)	
High	22	22.7% (9.7%–43.8%)	
VSR			0.0089
Low	22	31.8% (16.1%–52.8%)	
High	22	0% (−2.6%–17.5%)	

Abbreviations: BMI, body mass index; CI, confidence interval; FIGO, International Federation of Gynecology and Obstetrics; ORR, overall response rate; PLD, pegylated liposomal doxorubicin; SATA, subcutaneous adipose tissue area; VATA, visceral adipose tissue area; VSR, visceral to subcutaneous adipose tissue area ratio.

^a^ Others included six cases of clear cell, two cases of mixed, and three cases of undifferentiated carcinoma histology.

### 3.3 Survival and prognostic analyses

**FIGURE 2:**
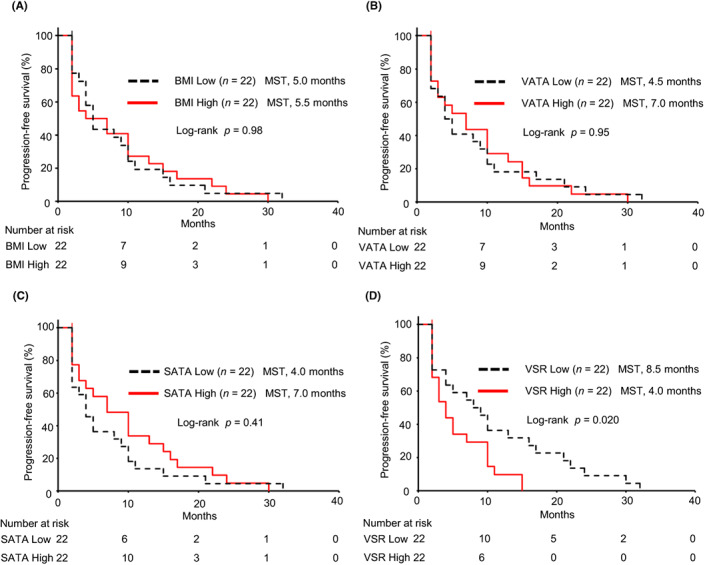
Kaplan–Meier curves of progression‐free survival. Patients were categorized into high and low groups according to the median values of BMI, VATA, SATA, and VSR. There were no significant differences between groups with high and low BMI (A, *p* = 0.98), VATA (B, *p* = 0.95), and SATA (C, *p* = 0.41). Patients in the high VSR group showed significantly shorter progression‐free survival compared with the low VSR group (D, *p* = 0.020). BMI, body mass index; MST, median survival time; SATA, subcutaneous adipose tissue area; VATA, visceral adipose tissue area; VSR, visceral to subcutaneous fat area ratio.

**FIGURE 3:**
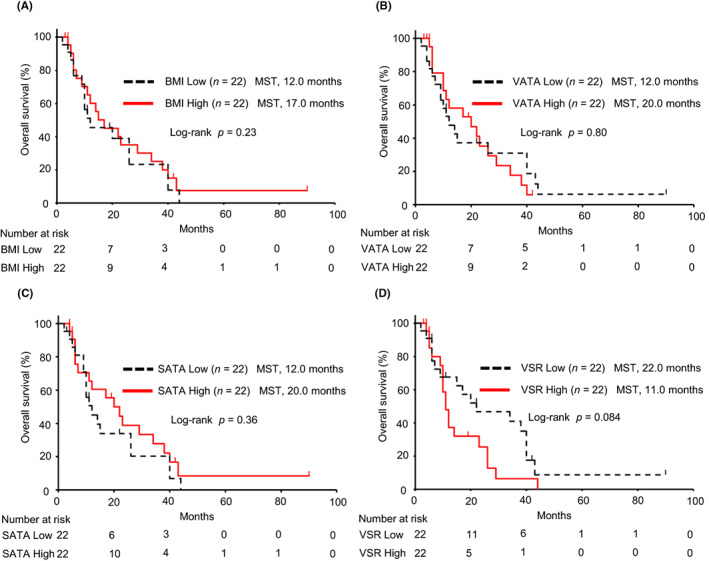
Kaplan–Meier curves of overall survival. Patients were categorized into high and low groups according to the median values of BMI, VATA, SATA, and VSR. There were no significant differences between groups with high and low BMI (A, *p* = 0.23), VATA (B, *p* = 0.80), and SATA (C, *p* = 0.36). There was a trend toward shorter overall survival in patients in the high VSR group compared with the low VSR group, but the difference was not significant (D, *p* = 0.084). BMI, body mass index; MST, median survival time; SATA, subcutaneous adipose tissue area; VATA, visceral adipose tissue area; VSR, visceral to subcutaneous fat area ratio.

We examined the association of adipose tissue areas and BMI with survival by log‐rank test. For PFS, Kaplan–Meier curves showed no significant differences between groups with high and low BMI, VATA, and SATA values (*p* = 0.98, *p* = 0.95, and *p* = 0.41, respectively; Figure 2A–C). However, patients in the VSR‐high group had significantly shorter PFS compared with the VSR‐low group (median, 4.0 vs. 8.5 months; *p* = 0.020; Figure 2D). For OS, no significant differences were found between the high and low BMI, VATA, and SATA groups (*p* = 0.23, *p* = 0.80 and *p* = 0.36, respectively; Figure 3A–C). OS tended to be worse in the VSR‐high patient group than in the VSR‐low patient group, but the difference was not significant (median, 11.0 vs. 22.0 months; *p* = 0.084; Figure 3D).

We also performed a multivariable analysis of survival with Cox proportional hazards model *adjusted by confounders*. As shown in Table 3, only high VSR was a significant prognostic factor for shorter PFS (HR, 2.07; 95% CI, 1.05–4.19; *p* = 0.035; Table 3). For OS, VSR showed a trend toward being a poor prognostic factor, although this was not statistically significant (HR, 2.02; 95% CI, 0.97–4.31; *p* = 0.060; Table 3). Table 4 shows the patients' characteristics for the high and low VSR groups. There were no significant differences in the clinical backgrounds of the two groups. Administered cycles of PLD were a median of 4.0 cycles (IQR 3.0–8.0 cycles) for the VSR‐high group and 8.0 cycles (IQR 3.0–18.0 cycles) for the VSR‐low group. Comparing the high and low VSR groups using the median number of administered cycles in all patients (5.0 cycles) as the cutoff value, no significant differences were found, as shown in Table 4. On the contrary, the administered cycles of PLD for the other groups were 5.5 cycles (IQR 3.0–10.5 cycles) for the BMI high group, 5.0 cycles (IQR 3.0–9.5 cycles) for the BMI low group, 5.5 cycles (IQR 3.0–12.8 cycles) for the VATA high group, 4.5 cycles (IQR 3.0–8.3 cycles) for the VATA low group, 7.0 cycles (IQR 3.0–13.0 cycles) for the SATA high group, and 4.0 cycles (IQR 3.0–8.3 cycles) for the SATA low group. Same as for the high and low VSR groups, no significant differences in the patients' characteristics were found between the high and low BMI, VATA, and SATA groups (data not shown).

**TABLE 3:** Adjusted hazard ratios of PFS and OS.

PFS			OS			
	Adjusted HR^a^	95%CI	*p* value	Adjusted HR^a^	95%CI	*p* value
BMI			0.77			0.60
Low (*n* = 22)	1	Reference		1	Reference	
High (*n* = 22)	0.90	0.44–1.83		0.81	0.36–1.79	
VATA			0.80			0.71
Low (*n* = 22)	1	Reference		1	Reference	
High (*n* = 22)	0.92	0.46–1.80		1.16	0.54–2.49	
SATA			0.18			0.44
Low (*n* = 22)	1	Reference		1	Reference	
High (*n* = 22)	0.61	0.29–1.26		0.73	0.32–1.62	
VSR			0.035			0.060
Low (*n* = 22)	1	Reference		1	Reference	
High (*n* = 22)	2.07	1.05–4.19		2.02	0.97–4.31	

Abbreviations: BMI, body mass index; CI, confidence interval; HR, hazard ratio; OS, overall survival; PFS, progression free survival; SATA, subcutaneous adipose tissue area; VATA, visceral adipose tissue area; VSR, visceral to subcutaneous adipose tissue area ratio.

^a^ Adjusted for age (<65 or ≥65), performance status (0 or ≥1), stage (I/II or III/IV), histological type (serous/endometrioid or others), previous chemotherapy regimens (≤2 or >2), platinum status (refractory or resistant).

**TABLE 4:** Characteristics of patients by VSR category.

Characteristic	VSR low (*n* = 22)	VSR high (*n* = 22)	*p* value
Age			0.76
<65	12 (55%)	13 (59%)	
≥65	10 (45%)	9 (41%)	
ECOG PS			1.00
0	22 (100%)	21 (95%)	
≥1	0 (0%)	1 (5%)	
FIGO stage			1.00
I/II	4 (18%)	4 (18%)	
III/IV	18 (82%)	18 (82%)	
Histological type			0.49
Serous/endometrioid	18 (82%)	15 (68%)	
Others^a^	4 (18%)	7 (32%)	
Residual disease			1.00
<1 cm	17 (77%)	17 (77%)	
≥1 cm	5 (23%)	5 (23%)	
Administered cycles of PLD			0.23
<5	8 (36.4%)	12 (55%)	
≥5	14 (63.6%)	10 (45%)	
Previous chemotherapy regimens			0.76
≤2	13 (59%)	12 (55%)	
>2	9 (41%)	10 (45%)	
Platinum status			0.76
Refractory	10 (45%)	12 (55%)	
Resistant	12 (55%)	10 (45%)	

Abbreviations: ECOG PS, Eastern Cooperative Oncology Group performance status; FIGO, International Federation of Gynecology and Obstetrics; VSR, visceral to subcutaneous fat area ratios.

^a^ Others included six cases of clear cell, two cases of mixed, and three cases of undifferentiated carcinoma histology.

## 4 DISCUSSION

The establishment of effective and safe treatments for patients with platinum‐refractory/resistant EOC is an urgent issue in gynecologic oncology. PLD is among the most commonly used anticancer agents in this patient group. Because PLD is effective in a minority of patients with platinum‐refractory/resistant EOC, there is a pressing need to discover novel biomarkers that predict which patients will respond to PLD treatment. This retrospective cohort study showed that a high VSR is associated with poor response and shorter PFS in patients with platinum‐refractory/resistant EOC treated with single‐agent PLD. Thus, VSR may be a useful biomarker for predicting the efficacy of PLD treatment.

There is currently a paucity of reports examining predictive biomarkers of PLD treatment. Ghisoni et al. showed that ovarian cancer patients with high expression of Type 2 topoisomerase alpha, which is involved in DNA replication and repair, have a higher response to PLD treatment.^28^ However, in this study, both platinum‐resistant and platinum‐sensitive patients were included, and some of the patients were received PLD in combination with other anticancer agents. In contrast, our study included only platinum‐refractory/resistant EOC patients who received single‐agent PLD, allowing us to evaluate the efficacy of PLD in a more homogeneous patient group. On the contrary, Dionísio de Sousa et al. observed that higher expression of low‐density lipoprotein receptor‐related protein 1B, which is one of the endocytic low‐density lipoprotein receptor superfamily, in PLD‐treated ovarian cancer patients was associated with prolonged PFS.^29^ However, more than 20% of the patients evaluated in this study had low‐grade serous carcinoma. Low‐grade serous carcinoma is a rare histological type, accounting for approximately 2% of EOC.^30^ Therefore, the patients' background in this study may be somewhat unbalanced. Furthermore, low‐grade serous carcinoma is originally resistant to chemotherapy,^30^ so it may be an inappropriate target for exploring biomarkers to predict the efficacy of PLD.

In the current study, we showed that VSR, an indicator of intra‐abdominal visceral adipose tissue accumulation, is a potential biomarker for predicting PLD efficacy in platinum‐refractory/resistant EOC patients. It has been reported that adipose tissue is associated with the efficacy of various anticancer agents. In breast cancer patients treated with neoadjuvant chemotherapy, patients with high VATA have significantly shorter disease‐free survival than those with low VATA.^14^ Additionally, in liver cancer patients who underwent transarterial chemoembolization, a lower response rate and significantly shorter PFS and OS were reported in patients with higher visceral adipose tissue density than in those with lower density.^15^

One possible mechanism by which adipose tissue influences the efficacy of anticancer agents is that it secretes a variety of growth factors, including angiogenic factors.^16^, ^17^, ^18^ Angiogenic factors derived from adipose tissue bind and neutralize the antiangiogenic agents, thereby attenuating their effects. Ladoire et al. studied the effect of antiangiogenic therapeutics in patients with renal cancer and reported that the time to progression or OS of patients with high VATA was shorter than that of patients with low VATA.^31^ We have also shown that high VATA is a predictor of poor response to bevacizumab, an antiangiogenic agent, in patients with recurrent EOC.^32^

Furthermore, adipose tissue can cause anthracycline resistance in cancer cells. Sheng et al. showed that adipose tissue absorbs and inactivates anthracyclines (daunorubicin and doxorubicin) through the expression of aldo‐keto reductases and carbonyl reductases, thereby inhibiting the effect of anthracyclines on leukemic cells.^19^, ^20^ Additionally, Lehuédé et al. showed that adipose tissue promotes extracellular efflux of doxorubicin by increasing MVP levels in breast cancer cells, thus inducing doxorubicin resistance.^21^ Because PLD is an anthracycline agent, it is presumed that there is an association between its efficacy and adipose tissue. However, there is a paucity of data regarding the relationship between PLD efficacy and adipose tissue. Herein, we provide the first report that shows adipose tissue modulates the efficacy of PLD in EOC patients and that adipose tissue can be a predictive biomarker for this chemotherapeutic agent.

In the current study, VSR was a biomarker for predicting the efficacy of PLD. It has been reported that VSR is a predictive biomarker of efficacy in a variety of cancer types and anticancer agents. In melanoma, higher VSR was correlated with decreased PFS and OS in patients who received antiangiogenic agents.^33^ In addition, high VSR has been associated with poor prognosis in liver, colorectal, and endometrial cancers.^24^, ^34^, ^35^ These reports suggest that high VSR is involved in resistance to anticancer agents and confers poor prognosis in cancer patients. However, the exact mechanism by which VSR engenders resistance to anticancer agents and poor prognosis is currently unknown. Visceral adipose tissue can promote cancer progression by inducing pro‐inflammatory adipokines such as tumor necrosis factor,^36^ which may partially explain why VSR is associated with resistance to anticancer agents and prognosis. However, further basic and clinical research is needed in order to confirm this hypothesis.

The current study has several limitations. First, it was single‐center and retrospective in design. Second, we excluded patients who had received two or fewer cycles of treatment with PLD. One reason for excluding such patients was that the tumor response to PLD was usually evaluated every three cycles at our institution. Patients who received two or fewer cycles of treatment were often not adequately evaluated for the tumor response. Furthermore, in these patients it was often unclear as to whether the reason for discontinuation of PLD was disease progression or poor general condition. For these reasons, we excluded patients who received two or fewer cycles of treatment from the study because of the difficulty in accurately assessing tumor response. Third, the cutoff value for VSR has not been clearly determined in general clinical practice thus far. In several previous reports, the cutoff value for VSR has often been taken as the median.^33^, ^34^ In the current study, the median value (0.44) was also used as the cutoff for VSR. Generally, a VSR value of 0.4 or higher increases the risk of heart disease and other adult diseases; thus, 0.4 is a commonly used cutoff value for visceral adiposity in health screening.^37^ The cutoff value for VSR in the current study was almost the same as the commonly used VSR cutoff value for visceral adiposity.

In summary, we have demonstrated that VSR can be a predictive biomarker of PLD efficacy in patients with platinum‐refractory/resistant EOC. Because these patients are often in poor general condition, predicting treatment efficacy is critical since it can help to avoid ineffective chemotherapy and onset of adverse events in these patients. In addition, CT scan is usually performed for the diagnosis of EOC recurrence, and hence, a new test for VSR measurement is not required. Therefore, the use of VSR as a biomarker is low‐cost, minimally invasive, and very useful in clinical practice. Further prospective studies are warranted.

## 5 CONCLUSIONS

A high VSR correlates with poor response and shorter PFS in patients with platinum‐refractory/resistant EOC treated with single‐agent PLD. VSR can be a reliable predictive biomarker for the efficacy of PLD. Measurement of VSR may provide useful clinical information to gynecologic oncologists when administering PLD to patients with platinum‐refractory/resistant EOC.

## AUTHOR CONTRIBUTIONS

**Hiroyuki Yoshida:** Conceptualization (lead); formal analysis (lead); writing – original draft (lead); writing – review and editing (lead). **Keiichi Fujiwara:** Supervision (supporting); writing – review and editing (supporting).

## CONFLICT OF INTEREST STATEMENT

The authors have declared no conflicts of interest.

## ETHICS STATEMENT

This study was approved by the Institutional Review Board of Saitama Medical University International Medical Center (reference number: 20‐096). This study was conducted in accordance with the Declaration of Helsinki.

## Data Availability

The data analyzed in the current study are available from the corresponding author on reasonable request.
